# Clinical and Immunological Phenotype of Patients With Primary Immunodeficiency Due to Damaging Mutations in *NFKB2*

**DOI:** 10.3389/fimmu.2019.00297

**Published:** 2019-03-19

**Authors:** Christian Klemann, Nadezhda Camacho-Ordonez, Linlin Yang, Zoya Eskandarian, Jessica L. Rojas-Restrepo, Natalie Frede, Alla Bulashevska, Maximilian Heeg, Moudjahed Saleh Al-Ddafari, Julian Premm, Maximilian Seidl, Sandra Ammann, Roya Sherkat, Nita Radhakrishnan, Klaus Warnatz, Susanne Unger, Robin Kobbe, Anja Hüfner, T. Ronan Leahy, Winnie Ip, Siobhan O. Burns, Manfred Fliegauf, Bodo Grimbacher

**Affiliations:** ^1^Department of Pediatric Pneumology, Allergy and Neonatology, Hannover Medical School, Hannover, Germany; ^2^Faculty of Medicine, Center for Chronic Immunodeficiency (CCI), Medical Center, University of Freiburg, Freiburg, Germany; ^3^Faculty of Biology, University of Freiburg, Freiburg, Germany; ^4^Faculty of Medicine, Center for Pediatrics, Medical Center-University of Freiburg, University of Freiburg, Freiburg, Germany; ^5^Laboratory of Applied Molecular Biology and Immunology, University of Tlemcen, Tlemcen, Algeria; ^6^Faculty of Medicine, Institute for Surgical Pathology, Medical Center-University of Freiburg, University of Freiburg, Freiburg, Germany; ^7^Cambridge Institute for Medical Research, Cambridge, United Kingdom; ^8^Acquired Immunodeficiency Research Center, Isfahan University of Medical Sciences, Isfahan, Iran; ^9^Department of Pediatric Hematology Oncology, Super Speciality Pediatric Hospital and PG Teaching Institute, Noida, India; ^10^Faculty of Medicine, Division Immunodeficiency (CCI), Department of Rheumatology and Clinical Immunology, Medical Center, University of Freiburg, Freiburg, Germany; ^11^Department of Pediatrics, University Medical Center Hamburg-Eppendorf, Hamburg, Germany; ^12^Infectious Disease Unit, Department of Medicine, University Medical Center Hamburg-Eppendorf, Hamburg, Germany; ^13^Department of Paediatric Immunology and Infectious Diseases, Our Lady's Children's Hospital Crumlin, Dublin, Ireland; ^14^Infection, Immunity and Inflammation Theme, UCL Great Ormond Street Institute of Child Health, London, United Kingdom; ^15^Department of Immunology, Great Ormond Street Hospital, London, United Kingdom; ^16^University College London Institute of Immunity and Transplantation London, United Kingdom; ^17^Department of Immunology, Royal Free London NHS Foundation Trust, London, United Kingdom; ^18^CIBSS-Centre for Integrative Biological Signalling Studies, University of Freiburg, Freiburg, Germany

**Keywords:** CVID, CID, NF-kappaB signaling, DAVID-syndrome, deficiency of anterior pituitary function and variable immunodeficiency, NF-kappaB2 clinical cases, ACTH-deficiency, autoimmunity

## Abstract

Non-canonical NF-κB-pathway signaling is integral in immunoregulation. Heterozygous mutations in *NFKB2* have recently been established as a molecular cause of common variable immunodeficiency (CVID) and DAVID-syndrome, a rare condition combining deficiency of anterior pituitary hormone with CVID. Here, we investigate 15 previously unreported patients with primary immunodeficiency (PID) from eleven unrelated families with heterozygous *NFKB2*-mutations including eight patients with the common p.Arg853^*^ nonsense mutation and five patients harboring unique novel C-terminal truncating mutations. In addition, we describe the clinical phenotype of two patients with proximal truncating mutations. Cohort analysis extended to all 35 previously published *NFKB2*-cases revealed occurrence of early-onset PID in 46/50 patients (mean age of onset 5.9 years, median 4.0 years). ACTH-deficiency occurred in 44%. Three mutation carriers have deceased, four developed malignancies. Only two mutation carriers were clinically asymptomatic. In contrast to typical CVID, most patients suffered from early-onset and severe disease manifestations, including clinical signs of T cell dysfunction e.g., chronic-viral or opportunistic infections. In addition, 80% of patients suffered from (predominately T cell mediated) autoimmune (AI) phenomena (alopecia > various lymphocytic organ-infiltration > diarrhea > arthritis > AI-cytopenia). Unlike in other forms of CVID, auto-antibodies or lymphoproliferation were not common hallmarks of disease. Immunophenotyping showed largely normal or even increased quantities of naïve and memory CD4^+^ or CD8^+^ T-cells and normal T-cell proliferation. NK-cell number and function were also normal. In contrast, impaired B-cell differentiation and hypogammaglobinemia were consistent features of *NFKB2*-associated disease. In addition, an array of lymphocyte subpopulations, such as regulatory T cell, Th17-, cTFH-, NKT-, and MAIT-cell numbers were decreased. We conclude that heterozygous damaging mutations in *NFKB2* represent a distinct PID entity exceeding the usual clinical spectrum of CVID. Impairment of the non-canonical NF-κB pathways affects function and differentiation of numerous lymphocyte-subpopulations and thus causes a heterogeneous, more severe form of PID phenotype with early-onset. Further characteristic features are multifaceted, primarily T cell-mediated autoimmunity, such as alopecia, lymphocytic organ infiltration, and in addition frequently ACTH-deficiency.

## Introduction

Common variable immunodeficiency (CVID [MIM 607594]) is a primary antibody deficiency syndrome defined by hypogammaglobulinemia, impaired production of specific antibodies, increased susceptibility to (primarily sinopulmonary) infections, and exclusion of secondary causes. The phenotype is complex, heterogeneous, and may include additional features, such as lymphoproliferation and autoimmunity. With an estimated prevalence of 1/10,000–50,000 CVID is the most common symptomatic primary immunodeficiency (PID) (1, 2).

Given that certain diagnostic criteria define CVID, it has been termed an “umbrella diagnosis” (3). The genetic basis of CVID is gradually being unraveled. Polygenetic inheritance is likely in most cases, but monogenic causes have been identified in 10–20% of affected individuals (3, 4). Importantly, monogenetic defects may represent distinct disease entities with particular phenotypes. Following identification in 2013 and 2015, heterozygous mutations in *NFKB1* and *NFKB2* now represent the largest CVID subgroups with known monogenetic mutations (5, 6).

NF-κB (nuclear factor kappa B) is key protein complexes regulating the immune response to infections. Their activation is mediated by two major pathways, the canonical NF-κB1 and non-canonical NF-κB2 pathway. The canonical pathway is stimulated by various immune receptors and primarily mediates rapid and broad inflammatory responses. In contrast, the non-canonical pathway is specifically stimulated and regulates lymphoid organ development, B cell maturation including germinal center reactions, T cell differentiation, thymic selection, and innate antiviral immunity (7–10). *NFKB2* encodes the cytoplasmic precursor p100, which preferentially dimerizes with RelB. Upon pathway stimulation p100 is phosphorylated and ubiquitinated at the C-terminal domain. Subsequently it is converted by proteasomal processing of its C-terminal half into the mature transcription factor subunit p52. Activated NF-κB dimers enter the nucleus and regulate target gene expression. Whereas transcriptional activation requires dimerization with one Rel subunit (which provides the transactivation domain), p52/p52 homodimers are transcriptional repressors. The hitherto reported C-terminal heterozygous mutations in humans disrupt the NF-κB-inducing kinase (NIK) mediated p100 phosphorylation (7–10). Subsequently, p100 processing to p52 is abolished. Thus, despite heterogeneity of the underlying mutation, those *NFKB2* mutations result in (functional) p52-haploinsufficiency.

Clinically, the first descriptions of patients affected by *NFKB2* mutations were characterized by a combination of CVID and ACTH insufficiency, a condition termed DAVID-syndrome (deficit in anterior pituitary function and variable immune deficiency) (11, 12). In addition, some patients have been described to suffer from various degrees of autoimmunity and trachyonychia (12–14). Since NF-κB signaling has a multitude of diverse functions within the immune system, the hitherto published phenotypic observations were highly heterogenic among the affected patients. Given the pivotal role of NF-κB in the immune system, it is conceivable that its dysregulation may cause a more severe type of early-onset PID, inflammatory-, autoimmune-, and malignant diseases exceeding the usual spectrum of CVID.

To elucidate this issue, we characterized a cohort of 15 novel patients and compared the phenotype with all 35 previously described patients with mutations in *NFKB2* (11–25). Our aim was the identification of putative genotype-phenotype correlations and common disease features, thus composing the current knowledge of the clinical and immunological phenotype in PID due to *NFKB2* mutations.

## Methods

### Patients

The study was reviewed and approved by the ethic commission of the Albert-Ludwigs Universität Freiburg, University of Freiburg, Germany, and informed and written consent for collection of patient history, clinical data, immunological studies, as well as for genetic analyses were obtained from the patients and their family members.

### Mutational Analysis in a CVID Patient Cohort by Targeted Next Generation Sequencing

Genetic analysis was performed in a large cohort of CVID patients as previously described (5). Briefly, genomic DNA was purified from PBMCs followed by Halo-Plex target enrichment according to the manufacturer's instructions (Agilent, Waldbronn, Germany). DNA samples were treated with a restriction-enzyme master mix and the products were hybridized to the HaloPlex probe capture library including the indexing primer cassettes. The target DNA was captured by a biotin-streptavidin system with HaloPlex magnetic beads, and the circular fragments were closed in a ligation reaction. The captured target libraries were amplified by PCR, and the amplified target libraries were purified with AMPure XP beads (Beckman Coulter) and washed in ethanol. Enrichment was validated on a BioAnalyzer or TapeStation (Agilent). Subsequently, samples were pooled in equimolar amounts for multiplexed sequencing on an Illumina MiSeq system. Libraries were denatured and diluted to a final concentration of 8–12 pM. For sequencing, an Illumina Reagent Kit v.2 was used and the following genes analyzed: *AKT1, APCS, BCL6, BLNK, BTK, CD19, CD27, CD274, CD28, CD40, CD40LG, CD79A, CD79B, CD80, CD81, CD86, CLEC16A, CR2, CTLA4, CXCL12, CXCR4, DCLRE1C, GATA2, ICOS, ICOSLG, IGHM, IGLL1, IKBKB, IKBKG, IL21, IL21R, IRF4, LRBA, MLH1, MS4A1, NFKB1, NFKB2, NFKBIA, PDCD1, PDCD1LG2, PIK3AP1, PIK3CD, PIK3R1, PRDM1, PRKCD, PRKD1, RAG1, RAG2, RELA, SH2D1A, STAT1, TCF3, TFRC, TGFB1, TGFB2, TGFB3, TNFRSF13B, TNFRSF13C, TNFRSF17, TNFRSF18, TNFRSF4, TNFSF10, TNFSF13, TNFSF13B, VAV1, VAV2*.

### Confirmation of Identified Sequence Variants by Sanger-Sequencing

Genomic DNA was isolated from blood samples using QIAamp kits (Qiagen, Hilden, Germany). Genomic fragments spanning exons 22 and 23 of *NFKB2* were amplified by PCR. PCR primers were used for Sanger sequencing according to standard techniques (sequences available on request). The frequency of the identified variations was analyzed with the databases SNPbase (http://www.ncbi.nlm.nih.gov/snp), 1,000 Genomes (http://browser.1000genomes.org/Homo_sapiens/Info/Index), EVS (http://evs.gs.washington.edu), Kaviar (http://db.systemsbiology.net/kaviar/), and ExAC (http://exac.broadinstitute.org/).

### NK and T Cell Assays

NK cell degranulation was performed as described (26). Briefly: Freshly isolated PBMCs were stimulated with either with medium alone or K562 cells (lacking MHC 1 expression) for 2.5 h in presence of anti-CD107a-PE (BD Biosciences, Heidelberg, Germany). Lytic exocytosis of NK cells (CD3- CD56+) are measured by CD107a (CD107a–PE (H4A3, IgG1) degranulation.

Cytotoxicity was measured by stimulating freshly isolated PBMCs by standard Chromium-51 release assay. K562 target cells were labeled with ^51^Cr and incubated with PBMCs. Supernatant was harvested after 4 h, transferred to lumaplates and dried overnight. Radioactivity was determined with TopCount NXT. NK cell percentage was measured by FACS staining and NK to target ratio was calculated. T cell proliferation was measured by CFSE dilution after 5–7 days of stimulation with medium alone or PHA (1.25 μg/ml) or CD3 (300 ng/ml) with CD28 (1,000 ng/ml) or CD3/CD28 Beads (BD Biosciences) as previously described (27).

### Circulating T Follicular Helper Cell Staining

Whole blood was stained with antibodies and fixed with Optilyse C (Beckman Coulter, Krefeld, Germany) according to the manufacturer's instructions. Data were acquired on a Gallios flow cytometer (Beckman Coulter) and analyzed using FlowJo software (version 10; Treestar, Ashland, OR, USA). Antibodies were: CD4 PerCP (clone SK3), CXCR5 FITC (clone RF8B2) (all from BD Biosciences, Heidelberg, Germany), CD3 PE-Cy7 (clone UCHT1, Beckman Coulter), CD45 Pacific Blue (clone HI30), CD45RA APC-Cy7 (clone HI100), CCR6 BV605 (clone G034E3) (all from BioLegend, San Diego, CA, USA), CXCR3 PE (clone WM59, R&D Systems, Minneapolis, MN, USA).

## Results

### Molecular Details of 15 PID Patients Due to Mutations in *NFKB2*

**Figure 1:**
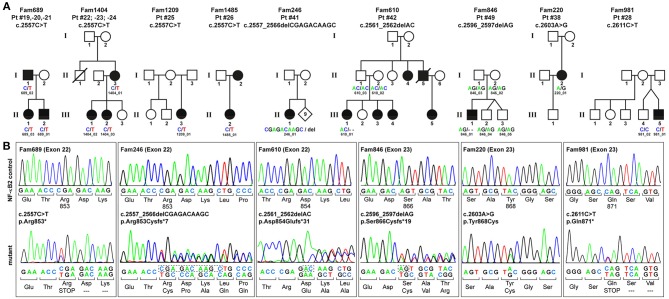
PID-associated C-terminal *NFKB2* mutations. **(A)** Pedigrees of the family members who participated in this study. Circles, female; squares, male; filled symbols, affected individual with PID; open symbol, healthy family member; slash, deceased individual. Family members who underwent genetic testing are indicated by IDs and sequence variants. **(B)** Sanger sequencing confirms that all *NFKB2* mutations cluster within exons 22–23 and affect the C-terminus of the 900 amino acids precursor protein p100. The mutation in Fam689 is shown as a representative for all families harboring the p.Arg853^*^ hot-spot mutation.

To identify the genetic origin of the disease phenotype in our CVID cohort we employed targeted Next Generation Sequencing (NGS). We identified 13 patients with *NFKB2* mutations affecting the C-terminal phosphorylation/ubiquitination domain (Figure 1).

Among these, eight patients from four different families carry the most common non-sense mutation in exon 22 (c.2557C>T; p.Arg853^*^), whereas further five patients harbor unique, previously unreported C-terminal mutations. All these mutations were confirmed by Sanger sequencing (Figure 1B). Despite the heterogeneity of the different mutations all previously described C-terminal mutations have universally been reported to result in normal expression and cytoplasmic localization of mutant, non-processable p100 precursors and thus in p52 haploinsufficiency (11–25) (Table 1).

**Table 1:**
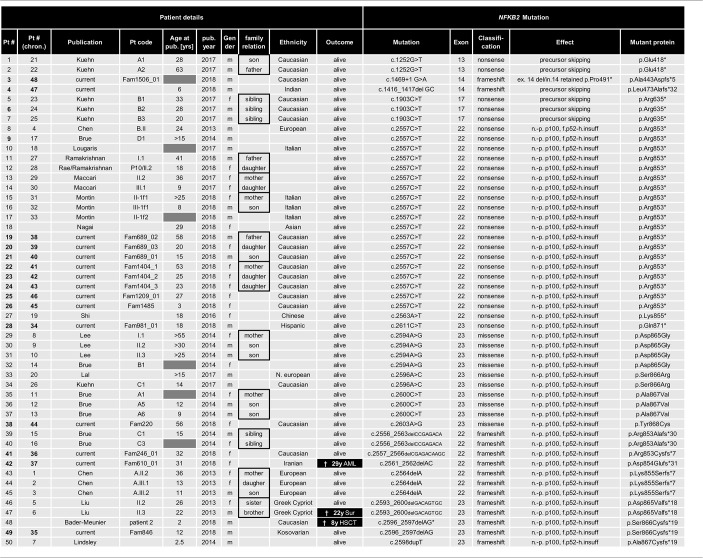
List of all 15 novel and 35 previously reported NFKB2 mutations.

*n.-p. p100, f.p52-h.insuff, non-processable p100 precursor functional p52 haploinsufficiency; AML, acute myeloid leukemia; Sur, surgery; HSCT, haemopoietic stem cell transplantation; ex, exon; in, intron; yrs, years. ^†^Deceased. Previously unpublished patient are indicated by bold writing*.

In addition to these C-terminal mutations, we identified two patients with proximal truncating mutations predicting the expression of severely shortened p52-like proteins, instead of the full-length p100, thereby skipping the precursor stage. The splice donor site mutation of intron 14 in patient P3 predicts splicing errors, such as skipping of exon 14 (resulting in a frame-shifted variant; p.Ala443Aspfs^*^5) or retention of intron 14 (resulting in a nonsense variant; p.Pro491^*^). However, we cannot exclude that other splicing errors occur. Likewise, the 2bp deletion in exon 14 (c.1416_1417delGC) in patient P4 predicts the premature termination of translation due to a shift of the reading frame (p.Leu473Alafs^*^32). Similar mutations have previously been described in five individuals (Table 1) (28). The novel mutations reported in the manuscript at hand have been functionally validated and will be published separately as the description of the molecular effects of these mutations is beyond the clinical and phenotypic scope of this manuscript.

### Case Descriptions of the Current Cohort

Previous studies indicated that the disease manifestations in NF-κB related diseases, even among mutations carriers within a single family, can be highly heterogeneous, ranging from asymptomatic status to severe PID phenotypes with recurrent, life-threatening infections. To identify common disease entities, and to enable comparative analyses with previously published cases, we reviewed patient records and describe the 15 PID patients with mutations in *NFKB2* from eleven different families participating in this study in detail. Patient aliases P1-P50 in the current study were assigned according to the position of the genetic variant within the *NFKB2* locus. The original patient identifiers as well as a chronological numbering of the published cases is also indicated (Table 1). The clinical phenotype is provided in Tables 2, 3, and summarized in Table 4. The immunological phenotype is summarized in Table 5, and detailed values of the immunological measurements are provided in Supplemental Table S1.

**Table 2:**
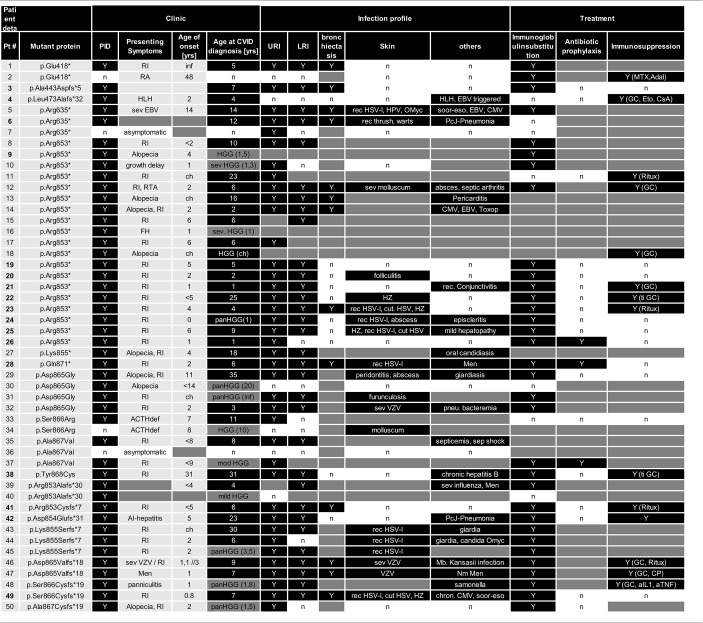
Clinical features, infection profile, and treatment of patients with *NFKB2* mutations.

*Adal, Adalimumab; absc, abscess; ACTHdef, ACTH-deficiency; AI, autoimmune; aIL1, anti-IL-1-therapy; ch, childhood; CP, cyclophosphamide; CMV, Cytomegaly Virus; cut, cutaneous; EBV, Epstein Barr virus; GC, glucocorticoids; HGG, hypogammaglobulinemia; HLH, hemophagocytic lymphohistiocytosis; HSV, herpes simplex virus; HSV-l, herpes labialis; HPV, human papilloma virus; HZ, herpes zoster; inf, infancy; Men, meningitis; mod, moderate; n, normal/negative (white); Nm, Neisseria meningitides; OMyc, onychomycosis; PcJ, Pneumocystis jirovecii; pneu, pneumococcal; rec, recurrent; RTA, renal tubular acidosis; RI, respiratory infections; Ritux, Rituximab; sev, severe; soor-eso, soor-esophagitis; SP, Streptococcus pneumonia; ti, topical/inhaled; Toxop, Toxoplasma gondii; trans, transitory; VZV, varicella zoster virus; Y, yes/present (black). No/insufficient information is indicated in dark gray*.

**Table 3:**
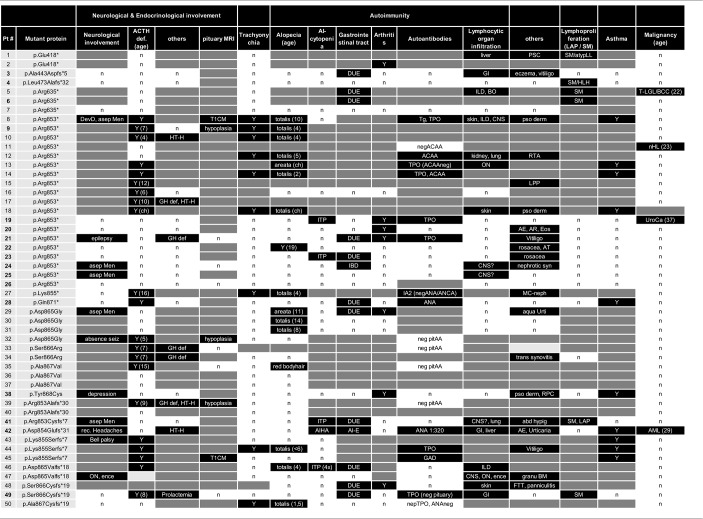
Clinical features, neurology, endocrinology, and autoimmunity in patients with *NFKB2* mutations.

*abd hypig, abdominal hyperpigmentation; ACAA, anti-cytokine autoantibodies; ACTHdef, ACTH-deficiency; AE, atopic eczema; AI-E-AI, enteropathy; AIHA, autoimmune hemolytic anemia; ANA, anti-nuclear antibodies; aqua Urti, aquagenic urticarial; AT, arterial hypertension; atypLL, atypical lymphoid hyperplasia; AI, autoimmune; BCC, basal cell carcinoma; BM, bone marrow; ch, childhood; CNS, central nervous system; DevD, developmental delay; DUE, diarrhea of unknown etiology; nHL-EBVneg, non-Hodgkin lymphoma (EBV neg); ence, encephalitis; Eos, eosinophilia; epid, epidermis; GAD, anti-Glutamat-Decarboxylase-antibodies; GI, gastrointestinal; gran BM, granulomatous disease of the bone marrow; GH, growth hormone; HT-H, Hypothyroidism; IA2, anti-Tyrosin-Phosphatase-autoantibody; ILD, interstitial lung disease; ITP, immune thrombocytopenia; inf, infancy; LAP, lymphadenopathy; LPP, lichen planus pigmentosus; MC-neph, minimal change nephropathy; mod, moderate; n, normal/negative (white); Nm, Neisseria meningitides; ON, optic neuritis; pita, pituitary autoantibodies; pneu, pneumococcal; OP, post-OP gastrointestinal bleeding; PSC, primary sclerosing cholangitis; pso derm, psoriasifrom dermatitis; rec, recurrent; RTA, renal tubular acidosis; seiz, seizures; sev, severe; SM, splenomegaly; T-LGLL, T-LGL leukemia; T1CM, type 1 chiari malformation; Tg, thyroglobulin; TPO, thyroperoxidase; UroCa, urothelial carcinoma; AR, allergic rhinitis; Y, yes/present (black). No/insufficient information is indicated in dark gray*.

**Table 4:** Summary of the major clinical manifestations of NF-κB2 haploinsufficiency.

**Patient characteristics**	**Total**	**Percentage**
	***n* = 50**	
**Sex ratio** (m/f)	27/23	
**GENOTYPE**		
Proximal mutations	7	
C-Terminal mutations	43	
Age at disease onset (mean; median (range) [years])	5.9; 4.0 (0.1–48)	
**CLINICAL MANIFESTATION**		
PID	46	92
**INFECTION PROFILE**		
URI	39	93
LRI	32	82
Bronchiectasis	13	57
Skin	19	61
Herpes disease	13	38
Candida disease	6	20
Other skin manifestations	7	20
Opportunistic infections/chon. CMV/EBV	8	16
Bac. Meningoencephalitis	3	6
Other severe bac. Infections	3	6
Giardiasis	3	6
**NEUROLOGICAL AND ENDOCRINOLOGICAL**		
Aseptic meningitis	5	10
Optic neuritis	2	4
ACTH deficiency	21	44
Other endocrin. manifestations	8	36
Other neurological manifestations	7	14
**Autoimmunity**	40	80
Trachyonychia	9	21
Alopecia	16	38
AI-cytopenia	5	10
Gastrointestinal manifestation	13	50
Arthritis	7	30
Autoantibodies	12	40
Lymphocytic organ infiltrations	15	52
CNS	6	
Lung	5	
Other organs	4	
Other autoimmune manifestations	17	65
**Lymphoproliferation**	6	32
Splenomegaly	6	32
Lymphadenopathy	2	10
**Malignancy**	4	8

*Percentages are provided as fraction of affected patients relative to the number of patients with information available*.

**Table 5:**
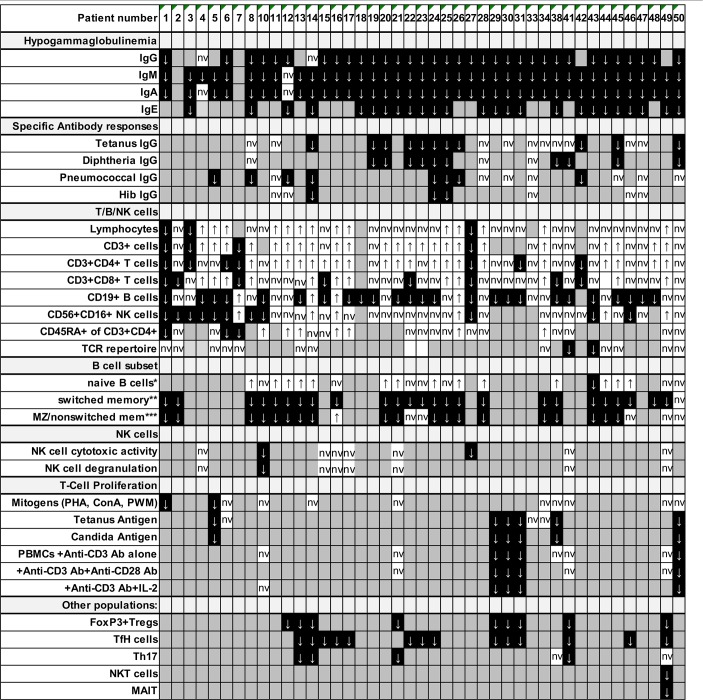
Immunological phenotype in patients with NFKB2 mutations.

Patient 1–7 carry proximal mutations, all other C-Terminal mutations. Black/arrow down, decreased; gray, no information provided; white nv, normal value; white/arrow up, increased; MZ, marginal zone. ^*^IgD^+^CD27^−^ of CD19^+^, ^**^IgD^−^CD27^+^, ^***^IgD^+^CD27^+^.

### Patients With C-Terminal Truncating Mutations Causing p52 Haploinsufficiency

**P19** (Fam689_02; father) suffered from recurrent respiratory tract infections starting at the age of 5 years. He was diagnosed with CVID at the age of 14 years when an autoimmune thrombocytopenia (ITP) occurred. Treatment was initiated with intravenous immunoglobulin replacement. The ITP which was chronic recurrent over a time period of 2 years was treated with oral steroid and pulses of intravenous immunoglobulins. At age of 37 years an urothelial carcinoma was diagnosed and cured. The patient is now 58 years old and besides suffering of mild arthritis alive and well.

**P20** (Fam689_03; daughter) suffered from infectious symptoms included recurrent respiratory tract infection, pneumonia, chronic purulent otitis media starting in her second year of life. In addition, allergic rhinitis and atopic dermatitis occurred. In line with this peripheral eosinophilia with values of 700–1,000 eosinophils/μl was observed. At the age of 2 years CVID was diagnosed. Treatment was limited to intravenous immunoglobulin replacement. Besides mild arthritis and recurrent folliculitis the patient is alive and well.

**P21** (Fam689_01; son) suffered from pneumonia at the age of 15 months and was subsequently diagnosed with PID due to family history. He developed rheumatoid factor-negative arthritis and diarrhea of unknown etiology recognized as immunological complication. Endocrinology revealed growth hormone deficiency and growth retardation. In addition, the patient suffered from vitiligo (Supplemental Figure S1) and recurrent conjunctivitis. Upon occurrence of epilepsy with an anxiety aura a cranial MRI was performed and showed no neuroanatomical pathology of the pituitary gland, brain stem, or hypothalamic region. Treatment was initiated with intravenous immunoglobulin replacement in early childhood, which was later switched to subcutaneous immunoglobulins. The arthritis was treated with NSAR and intra-articular steroid injections. The endocrinological treatment was commenced with GH-injections, and antiepileptic therapy with oxcarbazepine. Up to now, there are no signs of ACTH deficiency and the patient is alive and well.

**P22** (Fam1404_01, mother) is a female patient suffering from early childhood on from recurrent respiratory infections. Hair loss started at the age of 18. She had alopecia totalis at 19 years. Dermatologic findings were recurrent Herpes zoster and rosacea. Immunological work-up revealed CVID at age 25 and immunoglobulin substitution was initiated. Following 3 events of pneumonia during 1 year at 50, pulmonary work-up showed arterial hypertonia and small airways disease, but no signs of bronchiectasis. The patient is now 52 years old, alive and well under continuous immunoglobulin substitution.

**P23** (Fam1404_02, daughter) suffered from recurrent upper- and lower respiratory infections since early childhood on. Aged 4 years, she was diagnosed with CVID and immunoglobulin replacement was initiated. At 11 years she presented autoimmune thrombocytopenia treated with steroids and Rituximab. Chest-CT showed bilateral lower and middle lobes bronchiectasis. She is now 24 years-old with recurrent herpes labialis, cutaneous herpes infections, herpes zoster and rosacea. In addition, she suffers from episodes of diarrhea of unknown etiology.

**P24** (Fam1404_03, daughter) presented with neonatal pneumonia with full recovery. At the age of 1 year, hypogammaglobinemia was detected and IgG replacement therapy was initiated. Aged 13 years, she suffered from aseptic meningitis. At 16 years she had nephrotic syndrome, confirmed by renal biopsy (cause unknown). She had suffered from episcleritis, with two relapses; since last one no further complaints. She reported a soft tissue infection on the thumb, staphylococcus aureus was isolated. Influenza A virus infection was reported last year. In addition, she has recurrent labial herpes infections, upper respiratory infections around six times per year (which require antibiotic therapy) and chronic diarrhea of unknown etiology. Currently, she is alive and stable under immunoglobulin replacement therapy.

**P25** (Fam1209_01) is a female patient of 27 years presenting from childhood on with recurrent bronchitis and pneumonias. At the age of 6 years, she suffered from pneumonia and aseptic meningitis. During laboratory work-up hypogammaglobinemia was detected and immunoglobulin replacement started. She shows an elevation of liver enzymes from the age of 17 years until today with negative virology work-up. She suffers from recurrent HSV-1 outbreaks since childhood. Upon occurrence of genital herpes systemic therapy with acyclovir became necessary. She is alive, under immunoglobulin replacement therapy and acyclovir prophylaxis for recurrent herpes infections.

Female **P26** (Fam1485; daughter) was referred to immunology service at the age of 1 year after identification of low immunoglobulin levels following admission to high dependency unit for H1N1 infection, and family history of CVID in the mother (Pt. could not be included in this study). She had normal antenatal and neonatal period and first became unwell at 5.5 months of age when she was admitted for H1N1 infection requiring CPAP respiratory support. She was found to have low immunoglobulin levels and absent responses to tetanus and pneumococcal conjugate vaccines. The history of infections was otherwise unremarkable apart from one episode of otitis media that resolved with a course of oral antibiotics. Her mother was found to have hypogammaglobinemia at 18 months of age and had been on immunoglobulin replacement since. **P26** was started on immunoglobulin replacement and antibiotic prophylaxis with good effects. She has not experienced any other major infection since. She has mild eczema but nil else and is growing and developing well.

The male **P28** (Fam981_01) was conceived by *in vitro* fertilization and presented in early childhood with recurrent respiratory tract infections, pneumonia, otitis media, bronchitis, chronic sinusitis and diarrhea. CVID was diagnosed at the age of 6 years and immunoglobulin substitution initiated. Chest-CT showed bilateral lower lobe bronchiectasis. At the age of 11 years, he suffered of an episode of bacterial meningoencephalitis with full neurological recovery. Except for recurrent herpes labialis the patient is infection free under continued Ig-substitution and antibiotic prophylaxis. At age 16 hypopituitary central adrenal insufficiency was diagnosed. Treatment was commenced with replacement hydrocortisone.

Female **P38** (Fam220) suffered from recurrent respiratory infections during childhood, including pneumonia. Aged 31 years she was diagnosed with HBV-infection managed with interferon-therapy for 4 years. At this time, CVID was also diagnosed and immunoglobulin substitution was initiated. At 38 years, inhaled steroids were added to therapy due to intrinsic bronchial asthma. During follow-up she was diagnosed with psoriasis at age 42. She had several episodes of bronchitis, no season predominant; pathogens were not found in sputum. Bronchoscopy and BAL reported normal. She reported pinna, nose pain and arthritic ribs pain. ANCA levels negative. She developed saddle nose and tracheal stenosis and thus polychondritis was diagnosed. The patient is now 56 years old, alive, and suffering from mild depression, chronic liver damage (liver steatosis) and polychondritis.

**P41** (Fam246_01) was diagnosed with CVID at the age of 6 years with splenomegaly and autoimmune thrombocytopenia with a minimum platelet count of 8,000/μl. Infectious symptoms consisted at that time of a chronic obstructive bronchitis and a chronic sinusitis, present since early childhood. Respiratory work-up revealed bronchiectasis, obstructive ventilation disorder and signs of interstitial lung disease. At the age of 5 years, severe, aseptic meningitis developed. In addition, the patient suffers from diarrhea of unknown etiology. Lymphadenopathy prompted surgical resection and histological analysis showing only very small germinal centers (Supplemental Figure S2). Treatment was commenced with intravenous immunoglobulin replacement and steroids and rituximab for the autoimmune thrombocytopenia. Other family members refused genetic testing or providing health status information for personal reasons.

**P42** (Fam610_01) was diagnosed with CVID at the age of 17 years and additional immune-mediated diseases, such as autoimmune hepatitis and gluten-sensitive enteropathy. Infectious symptoms included recurrent respiratory tract infection, *Pneumocystis jirovecii* pneumonia, and diarrhea. Dermatology revealed recurrent urticaria and pruritis and eczematous lesions consistent with atopic dermatitis. As AP and y-GT were elevated and ANA was positive, a liver biopsy was performed consistent with autoimmune hepatitis. A duodenal biopsy showed a mild ileitis with increased intraepithelial lymphocytes in the terminal ileum and the right colon. Treatment was initiated with intravenous immunoglobulins and various immunosuppressive therapies including cyclosporine, tacrolimus, mycophenolate, and prednisolone. This treatment was stopped by the patient and later changed to intravenous immunoglobulins which provoked allergic reactions. During pregnancy at age 28 the clinical condition improved with neither infection, nor urticaria or pruritis, and also improved GI problems. A healthy baby girl was born with cesarean section due to due to pre-eclampsia. At the age of 29 the patient presented with fever and fatigue and was referred to a hematologist by G.P because of abnormal CBC. She was diagnosed with AML and died of severe aspergillus pneumonia after the first course of chemotherapy.

Starting in infancy, this male **P49** (Fam846_01) from Kosovo suffered from recurrent respiratory tract infections including pneumonia. In addition, he suffered from chronic sinusitis and chronic diarrhea since early childhood. At the age of 7 years, CVID, mild splenomegaly, and bilateral bronchiectasis were diagnosed, and immunoglobulin substitution initiated. The patient also suffered from recurrent herpes labialis and zoster at dermatomes Th9/Th10 at the age of 4 and 9 years. Blood analysis revealed a chronic CMV replication (4,000–10,000 copy numbers) without CMV-related organ complications. GI work-up showed mild soor-esophagitis and epithelial lymphocytic infiltrates in the GI-tract (Supplemental Figure S2). At the age of 8 years, ACTH-deficiency was diagnosed following a symptomatic hypoglycemia. Endocrinological work-up revealed chronic prolactinemia and cranial MRI showed no signs of hypophysitis microadenoma, or hypophysitis. Pituitary autoantibodies were negative. The patient is now 12 years old, alive, and well under continued cortisone- and immunoglobulin substitution.

### Patients With Proximal Truncating Mutations and Expression of p52-Like Proteins

**P3** (Fam1506) is a male patient who presented in early childhood with recurrent respiratory infections, otitis media, chronic sinusitis and pneumonia. CVID was diagnosed at the age of 7 years and immunoglobulin substitution was initiated. He presented with vitiligo and eczema during the follow-up. Chest CT showed bilateral bronchiectasis. He reported worsening productive cough during the last 18 months. *Streptococcus pneumoniae* was isolated from sputum. He has chronic diarrhea and extensive small bowel nodular lymphoid hyperplasia, but no evidence of malabsorption. He is alive, under treatment with prophylactic antibiotics, inhaled therapy, and immunoglobulin substitution.

**P4** was born to non-consanguineous Indian parents without significant family history and presented at 22 months of age with high grade fever and cytopenias. He had no rash or any apparent focus of infection. On clinical examination, he had normal height and weight, had no dysmorphic features. Besides massive hepatosplenomegaly he suffered from multilineage cytopenias, hypofibrinogenemia, hyperferritinemia, and histological evaluation of the bone marrow showed hemophagocytosis. EBV viral capsid antibody was positive, but there was no evidence of active EBV or CMV replication assessed by PCR. Perforin stain, NK cell degranulation and T cell cytotoxicity were normal excluding primary HLH. Due to the fulfillment of >5/8 HLH criteria, he was treated according to the HLH 2004 protocol, which was well-tolerated and showed rapid response. After discontinuation of all treatments the patient stayed well for now more than 2 years.

## Immunological Studies

### Decreased Lymphocyte Subpopulations in Patients With *NFKB2* Mutations

**Figure 2:**
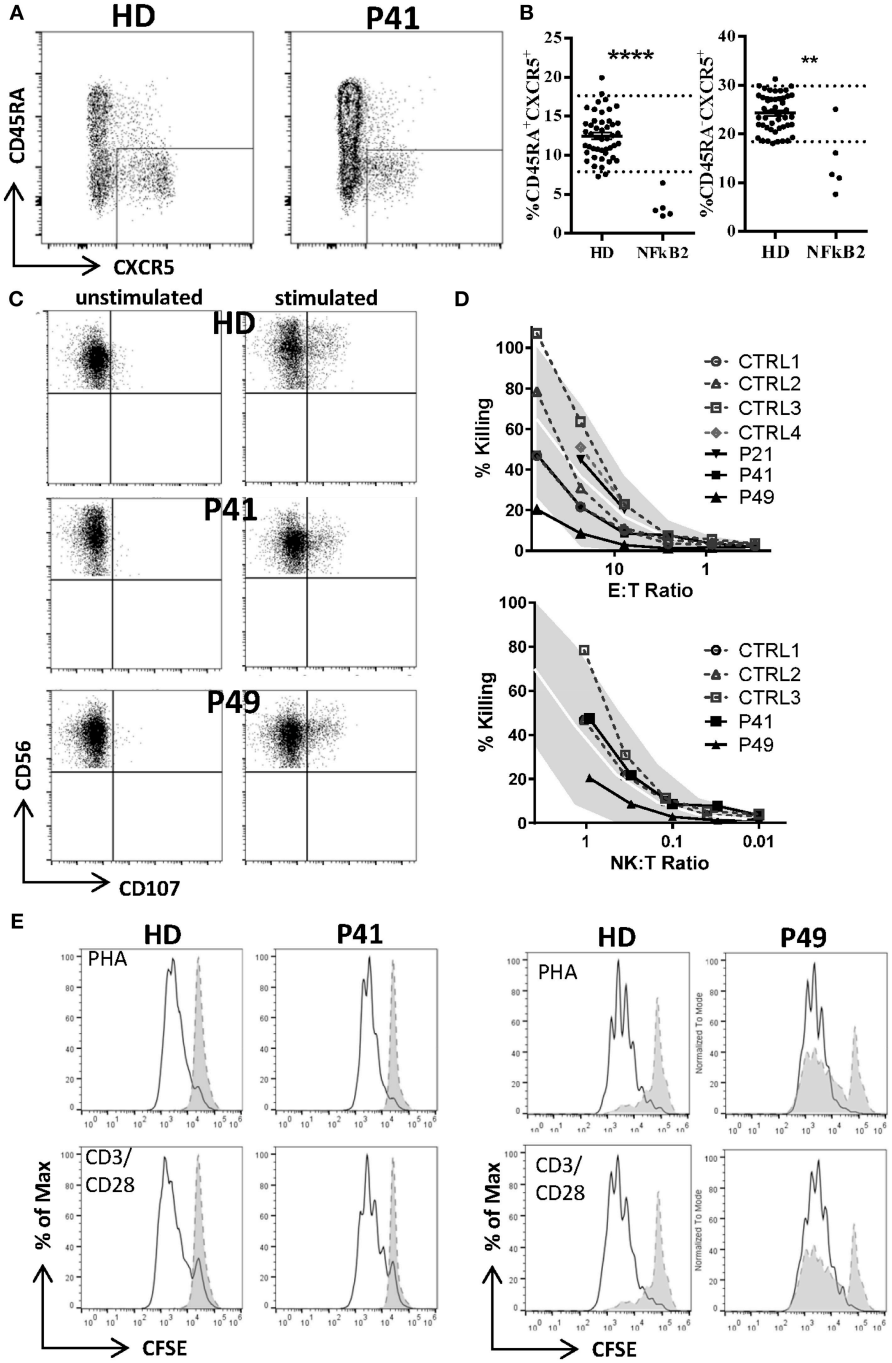
Decreased T follicular helper (cTFH), but normal NK and T cell function in *NFKB2*-mutated patients. **(A)** Reduced frequency of circulating T follicular helper cells (CXCR5posCD45RAneg) in CD4 T cells of patients with *NFKB2* mutation. FACS plots shows example staining of a healthy donor (HD) and P41. **(B)** Percentage of CXCR5pos in CD45RA+ (left) and memory (CD45RAneg, right) CD4 T cells. **(A,B)** Dotted lines in graphs represent 95% percentile of 47 healthy donors. Values of healthy donors are depicted as means ± SD. Statistical significance was assessed by Mann-Whitney test (**P < 0.01, ****P < 0.0001). **(C)** Normal NK and T cell function. **(C,D)** PBMCs were stimulated either with medium alone or K562 cells for 2.5 h in presence of anti-CD107a-PE. Lytic exocytosis of NK cells (CD56^+^) are measured by CD107a staining. **(D)** NK cytotoxicity was analyzed by standard 51Cr release assay. Target cell lysis by NK cells: PBMC were incubated with K562 target cells in a standard 51Cr release assay. The percentage of NK cells among PBMC was measured by flow cytometry to determine the NK:target cell ratio. The gray shaded area represents the range of normal values (5th−95th percentile). **(E)** T cell proliferation was measured by CFSE dilution after 1 week of stimulation with PHA (1.25 ug/ml) or CD3 (100 ng/ml) with CD28 (200 ng/ml).

Besides standard immunological diagnostics some patients underwent extensive immune-phenotyping. In P21 and P41 Th17 cells were found to be decreased as previously reported in other patients (P13, P14). However, Th17 cells in P49 were still within normal range (Table 5; Supplemental Table S1). FoxP3+ T regulatory T cells (Treg) have previously been described to be diminished (P12, P13, P14, P29, P30, P31) and were also decreased in our patients P41 and P49 who could be subjected to this assay. In line with previous reports, we observed reduced frequencies of circulating T follicular helper (cTFH) cells in CD4 T cells in patients (P22-24, P41, and P49) with C-terminal truncating *NFKB2* mutations (Figures 2A,B). However, when calculating the percentage of cTFH in memory CD4 T cells, P49 was in normal range, while the other four patients were below the normal range (Figure 2B), indicating that the reduction of circulating T follicular helper is not exclusive, but also a consequence of the diminished memory CD4 T cell compartment in this case. P49 could also be further assessed for NKT and CD161+Va7.2+CD8+ MAIT cells, which both were clearly decreased (Table 5; Supplemental Table S1).

### Normal Natural Killer–Cell Cytotoxic Activity in *NFKB2*-Mutated Patients

As one previous report indicated a defective natural killer–cell cytotoxic activity in *NFKB2*-mutated CVID patients we tested NK cell cytotoxicity as well as NK cell degranulation in P21, P41, P49. P21 and P41 demonstrate a completely normal NK function, but P49 showed normal NK degranulation and a mildly reduced NK function when assessing the NK:T ratio, while the E:T ratio was still normal (Figures 2C,D).

### Normal Ability of T Cells to Proliferate in *NFKB2*-Mutated Patients

Next, we addressed the question raised by previous reports whether NF-κB2 haploinsufficiency could impair T cell proliferation. However, T cells from all tested patients showed a normal response toward mitogens, such as PHA and CD3 ± CD28 stimulation as well despite different mutation classes (P21, P38, P41, P49) (Figure 2E). However, we could confirm a reduced proliferative response against candida antigen in P38.

In order to identify genotype-phenotype correlations and common disease features we compared our findings to all 15 previous publications reporting *NFKB2*-mutated patients.

### Clinical and Immunological Presentation in NF-κB2 Haploinsufficiency

Previous to this publication (as of Sep 1st 2018), 35 cases of *NFKB2* mutations have been reported, summing up with our novel 15 cases to a total of 32 families with 17 different mutations (Table 1). 47/50 mutation carriers are alive, the deceased patients died from a bleeding complication following abdominal surgery at age 22, aspergillus pneumonia after chemotherapy necessary due to AML at age 29 and complication of HSCT at the age of 8 years. In addition, two further deceased relatives of other patients (Brue et al.; Pt. C2, died at 9 years of age after BMT and Maccari et al.; Pt. I.2 deceased 68 years of age after CMV-colitis) are likely to have also been affected by mutations in *NFKB2*, but formal genetic testing was not possible and thus these cases have not been included in our cohort analysis (13, 20). All but seven reported *NFKB2*-mutated patients carry mutations in the C-terminal region, resulting in a non-processable p100 precursor protein and thus functional p52 haploinsufficiency (Table 1). The remaining reported four mutations cause proximal truncation predicting bypassing of the precursor but expression of mutant p52-like proteins (28).

### The Clinical Phenotype in NF-κB2 Haploinsufficiency is Characterized by Early-onset Antibody Deficiency, Clinical Signs of T Cell Dysfunction, and Autoimmunity Including Lymphocytic Organ Infiltrations

**Figure 3:**
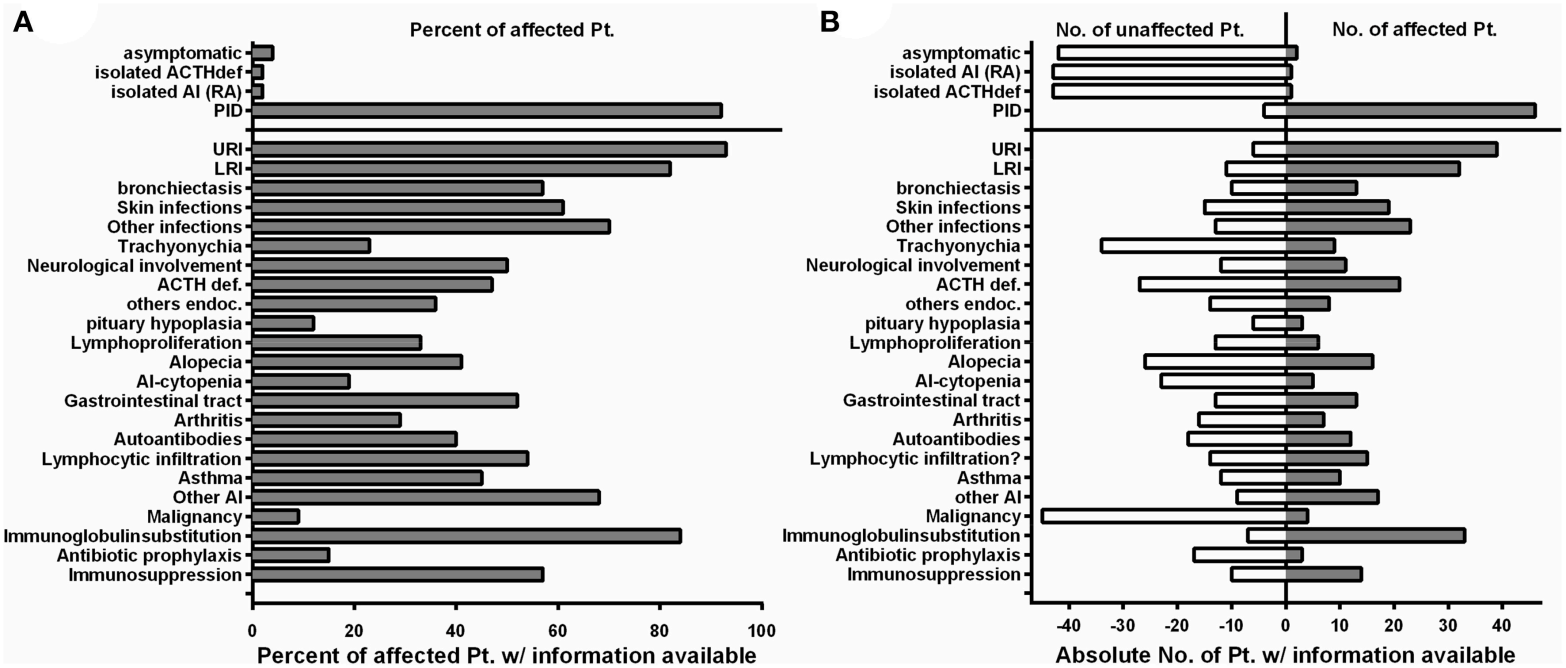
The clinical phenotype in NF-κB2 haploinsufficiency is characterized by early-onset antibody deficiency, clinical signs of T cell dysfunction, and autoimmunity. **(A)** Fraction of affected patients relative to the whole cohort of patients of whom information was available with different clinical manifestations depicted on the Y-axis. Manifestations below the solid line are relative to the 46 *NFKB2*-mutated patients with PID. **(B)** Absolute numbers of patients who are not affected by different clinical manifestations are depicted on the left (open white bars), while the absolute number of reported patients affected are depicted to the right side (solid gray bars).

PID was initially diagnosed in 46/50 of the affected individuals, which were later shown to harbor mutations in *NFKB2* (Figure 3; Table 4). Two of the non-PID individuals are asymptomatic, healthy *NFKB2* mutation carriers (P7, P36), whereas one individual suffers from isolated ACTH-deficiency only (P34), and one suffers from rheumatoid arthritis manifesting at age 48 (P2). Affected patients had a mean onset of symptoms of 5.9 years (SD ± 8.7; median 4.0 range 0.1–48 years). 26/45 (57%) patients with available information showed an onset of disease below the age of 5 years. The most common presenting symptom were respiratory infections (*n* = 30, 66%), followed by alopecia (*n* = 8, 18%). Other presenting symptoms were initial endocrinological abnormalities without initial signs of PID (3), severe herpes virus infection (2), and each one patient with meningococcal meningitis, nephritis, arthritis, secondary HLH, panniculitis, or autoimmune-hepatitis (Table 2).

In the PID group 33/46 (71%) patients were formally diagnosed with CVID (mean age 10.5; SD ± 9.1; median 6.5) and hypogammaglobinemia was noted in all the remaining patients (Table 2).

The infection profile demonstrates upper respiratory infections in 38/41 (93%) of the PID patients with available clinical information. Lower respiratory infections were described in 32/39 (82%) patients with bronchiectasis being reported in 13 of 23 (57%) cases with information available. Skin infections were reported in 19/31 (61%) patients, most commonly due to herpes species (13), but also bacterial causes (3), and severe molluscum (2) (Figure 3; Tables 2, 4). Candida esophagitis was noted in two patients, while four others suffered from susceptibility to skin/nail infections due to candida. Opportunistic infections included *Pneumocystis jirovecii* pneumonia in two patients, mycobacterial disease in one and chronic CMV viremia or EBV infections in three and two patients, respectively. Giardia was noted in three patients and salmonella in one. Three patients suffered from bacterial meningoencephalitis and other severe bacterial infections, such as septic arthritis or septicemia, were reported in three further patients. Further complications were pericarditis, toxoplasmosis, recurrent conjunctivitis or chronic hepatitis B each occurring in one patient each (Table 2).

ACTH-deficiency occurred in 21/48 (44%) mutation carriers with a medium manifestation age of 8.2 years (SD ± 4.2, median 7.0, range 4–15 years). All but two patients suffered also of PID preceding the occurrence of ATHC-deficiency (P32, P34). 8/22 (36%) of the patients showed other concomitant endocrinological abnormalities, with five growth hormone deficiencies, four hypothyroidism, and one child with prolactinaemia (Tables 3, 4). Pituitary auto-antibodies were negative in all 6 patients that have been subjected to this investigation. Cranial MRI showed pituitary hypoplasia in three, but normal pituitary findings in eight other affected patients. Interestingly, two unrelated patients showed type 1 chiari malformation as an incidental finding. Other neurological findings were single patient reports of developmental delay, epilepsy, absence seizures, bell palsy and depression. Interestingly, five patients were reported with aseptic meningitis, two with optic neuritis, and one with encephalitis (Table 3).

Concerning the autoimmunity in NF-κB2 haplodeficiency, 40 of 50 (80%) of patient were reported to suffer clinically from some form of autoimmune manifestation. Alopecia was present in 16/42 (38%) patients (12 alopecia totalis) with a mean manifestation age of 4.4 years (SD ± 5.1; median 4.0, range 2–19 years). Trachyonychia was noted in 9/43 (21%) mutation carriers (Figure 3; Table 3). The complete triad of trachyonychia, ACTH-deficiency, and alopecia occurred in 7 patients, and 10 patients showed at least two manifestations simultaneously (Table 3). Autoimmune cytopenias were reported in five patients, gastrointestinal manifestations in 12, and 7 patients suffered of arthritis. Interestingly, there was a surprisingly high number of patients with some kind of lymphocytic organ infiltration (15/29; 52%), mainly into the lung (5) and CNS (6). Other reported autoimmune manifestations were atopic eczema (3), psoriasifrom dermatitis (2), vitiligo (2), nephritis (2), rosacea (2), urticaria (2), as well as primary sclerosing cholangitis, lichen planus pigmentosus, panniculitis, and one case of granulomatous disease of the bone marrow. Autoantibodies, mainly TPO, have been found in 12 of 25 investigated patients. Asthma was reported in 10 patients (Figure 3; Table 3).

Information on lymphoproliferation was scarcely available but reported in 6/18 (33%) patients. Mild splenomegaly was the most common manifestation, lymphadenopathy was rarely noted. Lymph nodes were present in all of our patients; however, lymph node architecture was disturbed showing very small germinal centers in P41 (Supplemental Figure S2).

Four patients have developed a malignant disease: P5 suffered from T-LGL leukemia and basal cell carcinoma at age 22; P11 from an EBV-negative non-Hodgkin lymphoma at age 23; and P18 suffered from an urothelial carcinoma at age 37 and P41 suffered from AML at age 29 (Table 3).

Regarding treatment, 33/38 (84%) of PID patients receive immunoglobulin substitution, at least three patients received prophylactic antibiotics, and 13 patients required immunosuppressive treatment because of autoimmune manifestations (mainly steroids, but also 4 × anti-CD20 and 1 × cyclophosphamide as well as 1 × MTX and adalimumab) (Table 2).

### Preserved T/NK Cell Numbers and Function, But Disturbed B Cell Differentiation, Hypogammaglobulinemia and Decreased Lymphocyte Subpopulations Are Consistent Features of NF-κB2 Haploinsufficiency

**Figure 4:**
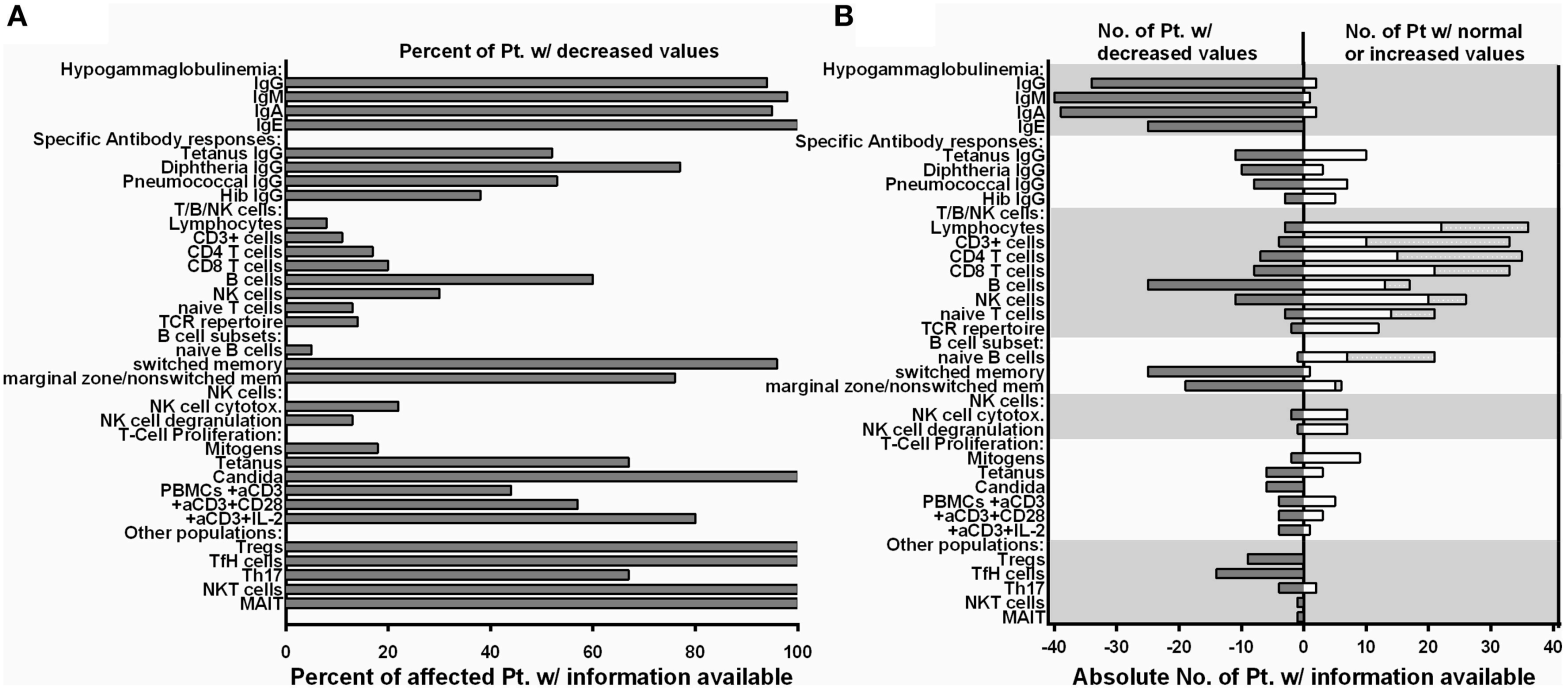
Preserved T/NK cell numbers and function, but disturbed B cell differentiation, hypogammaglobulinemia and decreased lymphocyte subpopulations are consistent features of NF-κB2 haploinsufficiency. **(A)** Fraction of affected patients relative to the whole cohort of patients of whom information was available regarding decreased values in the immunological phenotype depicted on the Y-axis. **(B)** Absolute numbers of patients with decreased values in different immunological assays are depicted on the left (solid gray bars), while the absolute number of patients with either normal or increased values are depicted to the right side (open white bars).

Detailed immunological data was available from 43 patients (Table 5; Figure 4; Supplemental Table S1). Hypogammaglobulinemia was consistently found in 34/36 (94%) patients for IgG, and 40/41 (98%) for IgM and IgA. No patient had increased values of IgE. Specific response to antigens were partially impaired with lack of titers against tetanus in 11/21 (52%), diphtheria 10/13 (77%), pneumococcus 8/15 (53%) and HiB 3/8 (38%) of cases. Only 3/39 patients were lymphopenic, while 22 had normal, and 14 had increased lymphocyte numbers. T cell numbers were low in 4/37, normal in 10 and elevated in 23 patients. The elevated T cell numbers were largely due to an expansion of CD4^+^ T cells (20/42). The fraction of naïve T cells was decreased in 3/24 (13%) patients contrasting 7 (30%) patients with increased percentages. TCR repertoire was normal in 12/14 (86%) investigations (Table 5; Figure 4).

B cell numbers were reported to be decreased in 25/42 (60%) reported cases. B cell differentiation proved to be impaired in most investigated patients with an increase of naïve IgD^+^CD27^−^ of CD19^+^ B cells in 14/22 (63%), reduced marginal zone/non-switched memory IgD^+^CD27^+^ cells in 19/25 (76%), as well as reduced IgD^−^CD27^+^ switched memory B cells in 25/26 (96%) (Table 5; Figure 4).

NK cell number was decreased in 11/37 (30%) patients. While two reports suggested decreased NK degranulation in one and slightly reduced NK cell cytotoxic activity in two patients (17, 18), another report of three other patients (carrying the same mutation) proved normal NK cell cytotoxicity and degranulation (25). In our hands, NK degranulation was normal in 3/3 further patients. Concerning NK-cytotoxicity, one patient with a frameshift mutation (P49) showed near-normal values, while two further patients (P21, P41) demonstrated fully functional NK-cytotoxicity (Figure 2).

Concerning T cell function, reduced T cell proliferation toward mitogens was reported in two patients, while 9 others had normal proliferative activity. TCR stimulation by anti-CD3 alone resulted in decreased proliferative response in 4/9 patients and could not be rescued by addition of anti-CD28. While reduced T cell proliferation against (antigen specific) TCR signaling was found in 6/6 investigated patients against candida, 3/9 other patients showed normal proliferative response toward tetanus (Table 5; Figure 4).

In all 9 investigated patients Foxp3^+^ Treg cells were decreased. as it was the case for all 14 patients investigated for T follicular helper cells. Th17 cells were decreased in 4/6 of the patients investigated. In addition, we tested a single patient for NKT and CD161^+^Va7.2^+^CD8^+^ MAIT cells, which proved to be reduced (Table 5; Figure 4).

## Discussion

Although both, canonical and non-canonical NF-κB signaling is of central importance for various aspects of immunoregulation, mutations in *NFKB1* and *NFKB2* have only very recently been recognized as a molecular origin of the disease phenotype in CVID. Here, we summarize the current knowledge of the immunodeficiency phenotype associated with mutations in *NFKB2*, in a cohort of 15 novel and 35 previously described patients.

### Clinical Presentation of Patients With Damaging *NFKB2* Mutations

Early-onset PID and a variable degree of autoimmunity became evident in the vast majority of individuals with mutations in *NFKB2* (Tables 2–4; Figure 3). ACTH-deficiency occurred in 44% of mutation carriers. In all but two patients this manifestation was preceded by PID symptoms. Thus, occurrence of immunodeficiency, mainly in terms of respiratory infections typical for antibody deficiency, was the major presentation of *NFKB2*-mutated patients before pituitary dysfunction became apparent in the further course of disease (Table 2; Figure 3). Besides immunodeficiency, autoimmunity in terms of alopecia was the second most common initial disease manifestation. Of note, nearly 60% of patients had an onset of disease before the age of 5 years. Only two *NFKB2* mutation carriers were completely asymptomatic, one further patient suffered from late-onset arthritis as the only manifestation (Table 2). This illustrates that clinical expressivity and penetrance of *NFKB2* mutations resulting in DAVID-Syndrome, PID- or isolated ACTH-deficiency are heterogenic and difficult to predict based on the genetic alteration. However, the here described cohort underlies a high selection bias, as patients were mainly recruited in immunology centers. Given the heterozygous nature of *NFKB2* related diseases and thus higher probability of spontaneous occurrence, future studies focusing on endocrinological cohorts are likely to identify further patients presenting with primarily pituitary dysfunction and lack of overt immunological abnormalities.

### Humoral Immunodeficiency in *NFKB2*-Mutated Patients

From a clinical point of view, impaired B cell differentiation, hypogammaglobulinemia, and subsequent susceptibility to respiratory infections were present in most patients and thus compatible with CVID (Figures 3, 4; Tables 2, 5). Interestingly, the ability of mounting a specific antibody response was at least partly preserved in more than 50% of the tested patients and thus is in conflict to one ESID diagnostic criterion of CVID (Figure 4; Table 5). However, marginal zone and switched memory B cells were reduced in all PID patients, while total B cell count was decreased in only about half of the affected patients (Figure 4; Table 5). This suggests that B cell function and differentiation, rather than their absolute number, play essential roles in pathogenesis of immunodeficiency in patients with *NFKB2* mutations. The pivotal role of the B cell intrinsic NF-κB2 pathway in humoral immune responses for B cell survival and maturation has been demonstrated in humans and murine models (29, 30). In addition, a cellular mechanism for the observed phenotype is offered by the reduction of cTFH-cells in p52 haploinsufficiency patients [Figures 2, 4; Table 5 and Ref. (16)]. cTFH-cells are considered to be the circulating counterparts of TFH in secondary lymphoid tissue (31). TFH cells are specialized providers of T cell help to B cells, and are essential for germinal center formation, affinity maturation, and the development of high-affinity antibodies and memory B cells (32). The non-canonical NF-κB pathway has been shown to play an important role in TFH development by regulating the expression of inducible T cell costimulator (ICOS) ligand in B cells, and thus p52 haploinsufficiency is likely to cause the disturbances of the humoral immune axis (32, 33). In conclusion, a fully functional NF-κB2 pathway is required for successful germinal center reactions by enabling survival and maturation of B cells and generation of TFH cells and thus p52 haploinsufficiency results in the observed humoral phenotype.

### More Than CVID: Immunodeficiency in Patients With Damaging *NFKB2* Mutations

Many patients affected by *NFKB2* mutations demonstrated a severe clinical course, which is not typical for isolated antibody deficiency (Figure 3; Table 2). In addition, 13 patients showed antibody deficiency before the fourth year of life which also refutes the definition of CVID by the ESID diagnostic criteria. A complicated disease course was associated with clinical signs of T cell dysfunction, such as the susceptibility to recurrent or severe herpes virus infections, candida infections, and severe opportunistic infections, such as *Pneumocystis jivorecii* pneumonia in two patients (Table 2). However, unlike in combined immunodeficiency (CID), CD8^+^, CD4^+^, and CD45RA^+^ naive T cell numbers were generally normal or even increased (Figure 4; Table 5; Supplemental Table S1). Functional testing showed normal T cell proliferation in our hands, while previous reports produced conflicting results with either impaired T cell proliferation (14, 15) or also normal function (Figures 2, 4; Table 5) (20, 28). As the NF-κB2 pathway is dispensable for the initial activation of T cells through TCR signaling and rather important for regulating differentiation, further studies in humans are needed to elucidate a possible impairment of T cell function (34). Likewise, we could not confirm previous findings of impaired NK degranulation or NK cell cytotoxic activity (17, 18), as both were normal or merely marginally decreased in our cohort and one further study (Figure 2) (25). Therefore, rather than severe impairment of NK cell function or generally CID-like impaired T cell numbers or function, we favor the hypothesis of a pathogen-specific involvement of the NF-κB2 pathway being causative for the observed clinical spectrum. For an instance, the marked and hitherto underappreciated susceptibility toward herpes viruses may be related to the crucial functions of CD27, CD40, and LTβR, in the defense against herpes viruses, all of which signal through p100/p52 (35, 36). On a cellular level, the observed reduction of Th17 cells in the majority of investigated patients may predispose to candida susceptibility (37). However, the precise role of individual NF-κB subunits in the differentiation of Th17 needs to be further studied, as the interplay between NF-κB and RORγt in Th17 cells is not yet clear and two patients demonstrated normal Th17 numbers. This finding is in line with a mouse study, showing that the non-canonical NF-κB pathway is dispensable for Th17 differentiation, but essential for production of inflammatory cytokines by this subpopulation (38). Correspondingly, studies in the cascade of impaired non-canonical NF-κB-signaling and differentiation of TFH, NKT, and MAIT cells will increase our understanding of lymphocyte differentiation. An also noteworthy observation was the development of HLH in infant P4. As NK-cell function was assessed to be normal also in this patient, this further illustrates our previous observation from 64 patients who developed secondary HLH in the context of different PIDs including (S)CID and lymphocyte activation defects (27, 39).

### Autoimmunity in Patients With *NFKB2* Mutations: Rather T Cell Mediated Than Caused by Auto-Antibodies?

Our cohort analysis revealed that most *NFKB2* mutation carriers suffered from variable and sometimes severe autoimmune phenomena (Figure 3; Table 3). Besides ACTH-deficiency, alopecia, various lymphocytic organ-infiltration, diarrhea, and arthritis were common.

It is currently uncertain whether the ACTH-deficiency now observed in 21/48 cases has an autoimmune origin. The initial hypothesis of an involvement of *NFKB2* mutations in pituitary development, as indicated by hypoplasia in cMRI in the first patient, cannot be supported anymore (11), as an increasing number of affected patients had normal cMRI findings (Table 2). Also, “Lym1”-mice carrying homozygous nonsense mutations in *Nfkb2*, deleting the NIK-responsive phosphorylation domain (*Nfkb2*^Lym1/Lym1^; c.2854A>T; p.Tyr868^*^) show similar B cell findings as patients with *NFKB2* mutations but normal pituitary anatomy (13). However, no autoantibodies against pituitary proteins could be detected in all investigated patients. Likewise, autoimmune-cytopenias, which usually account for 2/3 – 3/4 of all autoimmune manifestation in other CVID patients (2), occurred only in 5/50 patients in the *NFKB2* cohort. Mechanistically, aberrantly activation of the NF-κB2 pathway promotes prolonged B cell survival and renders self-reactive B cells more resistant to negative selection, causing the generation of autoantibodies in other systemic autoimmune diseases (40). Altogether, this argues against a substantial production of auto-antibodies in NF-κB2 haploinsufficient patients. Whether the observed ACTH-deficiency is caused by T cell mediated autoimmunity instead is currently unknown, but the frequent presentation of lymphocytic organ infiltrations in half of the reported cases, including infiltration of the CNS in six patients, supports this hypothesis (Table 3).

Alopecia, predominately occurring early around the fifth year of life, was a very common manifestation with 16/50 affected patients and recognized as initial disease manifestation before apparent signs of immunodeficiency in half of them. Alopecia is understood to be a T cell dependent autoimmune disease (41). Interestingly, NKT cells have recently been demonstrated to be protective in alopecia. Thus, our single patient observation of reduced NKT cells in addition to previous reports demonstrating the importance of the NF-κB2 pathway for the development of NKT cells (42), may offer an explanation for the high incidence of alopecia in our cohort. Interestingly, trachyonychia, a distinct form of nail dystrophy observed frequently in *NFKB2*-mutated patients but also other diseases, is *per se* associated with alopecia and mild to moderate lymphocytic infiltration (43). Therefore, trachyonychia may facilitate another T cell mediated form of an autoimmune manifestation in *NFKB2*-mutated patients.

An additional explanation for the distinct profile of autoimmune manifestations observed in NFKB2-mutated patients is likely to be caused by the disturbance of central tolerance. NF-κB2 signaling is crucial for the development of medullary thymic epithelial cells (mTEC), which mediate immune tolerance by eliminating autoreactive T cells and promote generation of Tregs cells (44). In addition, NF-κB2 signaling regulates the expression of autoimmune regulator (AIRE). AIRE is a transcription factor in mTEC required for expression of self-tissue antigens in the thymus, necessary for the elimination of autoreactive T cells and induction of central tolerance (44, 45). Mutations in AIRE results in autoimmune-polyendocrinopathy-candidiasis-ectodermal dystrophy (APECED) in which self-reactive T cells occur and alopecia is a common feature in about 40% of cases (45). Further support for the hypothesis of disturbed central tolerance due to AIRE dysfunction in p52 haploinsufficiency is derived from the murine Lym1 model, which also has reduced thymocyte AIRE expression (46). In addition to the Addison-related hyperpigmentation, the major skin manifestations of APECED include chronic mucocutaneous candidiasis, nail dystrophy, alopecia areata or totalis, and vitiligo (45), which collectively closely resemble clinical aspects of p52 haploinsufficiency (Table 3; Figure 3; Supplemental Figure S1). As a previously underappreciated high number of *NFKB2*-mutated patients further suffered from lymphocytic organ infiltrations, the hypothesis of disturbed central tolerance due to *NFKB2* mutations and primarily T cell mediated autoimmune processes seems likely. Along this line of arguments (visceral) lymphocytic infiltrations were also observed in *Nfkb2*^Lym1/Lym1^ mice (46). A further explanation for the high incidence of autoimmunity is offered by the reduced number of regulatory T cells in all patients investigated (Figure 4; Table 5). This finding may be explained by the recent discovery of NF-κB activation being critical for thymic Treg development aided by mTEC as discussed above (44, 47).

### Conclusion

We conclude that heterozygous damaging mutations in *NFKB2* represent a distinct PID entity exceeding the usual clinical spectrum of CVID. Impairment of the non-canonical NF-κB pathway affects pivotal mechanisms of central tolerance as well as function and differentiation of numerous lymphocyte-subpopulations. Therefore, damaging *NFKB2* mutations cause a more severe form of PID with early-onset and a distinct, multifaceted auto-immunity with primarily T cell mediated autoimmune diseases, such as alopecia, lymphocytic organ infiltration, and possibly ACTH-deficiency.

## Author Contributions

CK cared for patients, performed literature research, collected clinical information, analyzed data, designed layout, figures and tables, and wrote the manuscript. NC-O collected clinical information and laboratory values, summarized data, and wrote case reports. LY performed experiments Fam220. ZE organized blood sample collection of the Iranian family and accomplished shipment. JR-R performed DNA extractions and Sanger re-sequencing. NF performed next generation sequencing and data evaluation. AB evaluated NGS and WGS data. MH cared for patients, provided clinical and immunological information, and wrote a case report. MA-D performed Sanger sequencing. JP reviewed medical records. MS contributed histological analyses. SA performed immunological studies in Pt. 41&49. RS cared for patients, provided clinical and immunological information, and wrote a case report. NR cared for patients, provided clinical and immunological information, and wrote a case report. KW cared for patients, provided clinical and immunological information, and wrote a case report. SU performed cTFH analysis. RK cared for patients, provided clinical and immunological information, and wrote a case report. AH cared for patients, provided clinical and immunological information, and wrote a case report. TL cared for patients, provided clinical and immunological information, and wrote a case report. WI cared for patients, provided clinical and immunological information, and wrote a case report. SB cared for patients, provided clinical and immunological information, and wrote a case report. MF performed NFkB2 experiments prepared figures and wrote the manuscript. BG coordinated the study and revised the manuscript. All authors commented on the manuscript.

### Conflict of Interest Statement

The authors declare that the research was conducted in the absence of any commercial or financial relationships that could be construed as a potential conflict of interest.
