# Supplementary material for: Clinical and Immunological Phenotype of Patients With Primary Immunodeficiency Due to Damaging Mutations in NFKB2
Source: Front Immunol. 2019 Mar 19;10:297. doi: 10.3389/fimmu.2019.00297 (PMC6435015; doi:10.3389/fimmu.2019.00297)
Supplement: Supplementary file 2 [file Data_Sheet_1.docx]

# Supplemental Data

# Supplement Table

## Legend Supplement Table S1:

**Supplement Table S1. Immunological phenotype in patients with *NFKB2* mutations.** Black/ arrow down=decreased, grey=no information provided, white nv=normal value, white/arrow up=increased. MZ=marginal zone *IgD+CD27- of CD19+, **IgD-CD27+, ***IgD+CD27+.

## Supplemental Figure S1


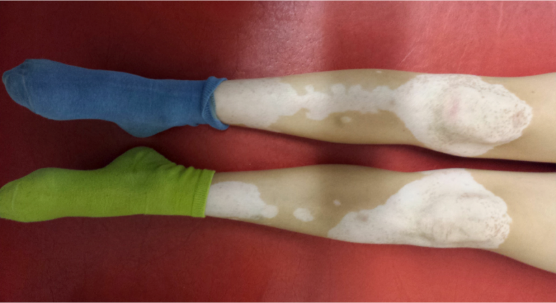


**Supplemental Figure S1.** **Vitiligo in Patient P2 (Fam689).** The photograph demonstrates the pronounced vitiligo patient P21 is suffering from.

## Supplemental Figure S2

##
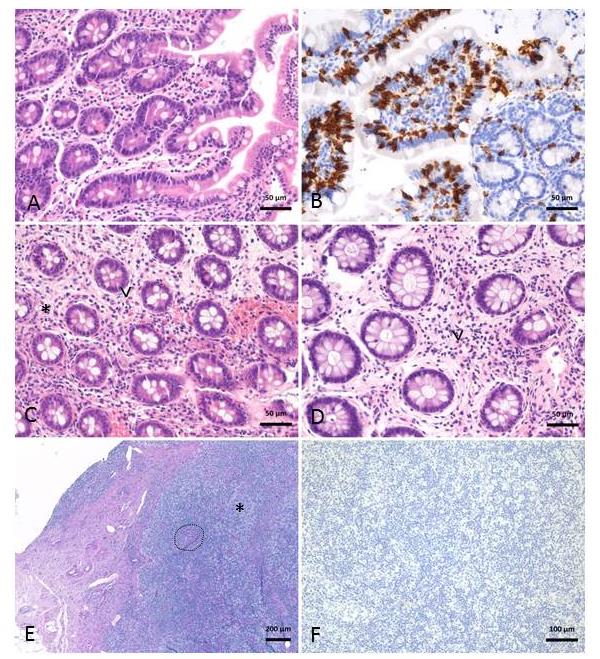


**Supplemental Figure S2.** Histology of *NFKB2*-mutated patients. **(A-D)** P49 (Fam846). **(A)** Duodenal biopsy with normal architecture and without inflammation but lacking stromal plasma cells. **(B)** CD8 staining (brown) shows villi with high numbers of intraepithelial lymphocytes. **(C, D)** Biopsies from the colon descendens **(C)** and rectum **(D)** display minor chronic damage with few apoptotic bodies in the stroma (C; asterisk, exemplary), patchy eosinophilic infiltrate (C, D; arrowhead) and reduced crypt diameters. **(E, F)** P41 (Fam246). **(E)** Lymph node with sparse, small germinal centers (dotted line, exemplary) and cortical macrophage infiltration (asterisk, exemplary) and lacking plasma cells in the CD138 staining **(F)** Original magnifications indicated by scale bars.
